# Impact of center volume on outcomes in allogeneic hematopoietic cell transplantation for children

**DOI:** 10.1038/s41409-025-02569-3

**Published:** 2025-04-10

**Authors:** Motohiro Kato, Hideki Nakashone, Keitaro Matsuo, Yuri Ito, Atsumi Yanagisawa, Marie Ohbiki, Ken Tabuchi, Tatsuo Ichinohe, Yoshiko Hashii, Junya Kanda, Hideki Goto, Koji Kato, Makoto Yoshimitsu, Atsushi Sato, Moeko Hino, Kimikazu Matsumoto, Kimikazu Yakushijin, Yoshiko Atsuta, Takahiro Fukuda

**Affiliations:** 1 Department of Pediatrics, The University of Tokyo, Tokyo, Japan; 2 Division of Hematology, Jichi Medical University Saitama Medical Center, Saitama, Japan; 3 Division of Emerging Medicine for Integrated Therapeutics, Center for Molecular Medicine, Jichi Medical University, Shimotsuke, Japan; 4 Division Cancer Epidemiology and Prevention, Aichi Cancer Center Research Institute, Nagoya, Japan; 5 Department of Medical Statistics, Research & Development Center, Osaka Medical and Pharmaceutical University, Takatsuki, Japan; 6 Japanese Data Center for Hematopoietic Cell Transplantation, Nagakute, Japan; 7 Department of Hematology and Oncology, Research Institute for Radiation Biology and Medicine, Hiroshima University, Hiroshima, Japan; 8 Department of Pediatrics, Osaka International Cancer Institute, Osaka, Japan; 9 Department of Hematology, Graduate School of Medicine, Kyoto University, Kyoto, Japan; 10 Division of Laboratory and Transfusion Medicine, Hokkaido University Hospital, Sapporo, Japan; 11 Department of Hematology, Oncology and Cardiovascular Medicine, Kyushu University Hospital, Fukuoka, Japan; 12 Department of Hematology and Rheumatology, Graduate School of Medical and Dental Sciences, Kagoshima University, Kagoshima, Japan; 13 Department of Hematology and Oncology, Miyagi Children’s Hospital, Sendai, Japan; 14 Department of Pediatrics, School of Medicine, Chiba University, Chiba, Japan; 15 National Center for Child Health and Development, Tokyo, Japan; 16 Department of Medical Oncology and Hematology, Kobe University Hospital, Kobe, Japan; 17 Department of Hematopoietic Stem Cell Transplantation, National Cancer Center Hospital, Tokyo, Japan

**Keywords:** Paediatrics, Haematological diseases

## Abstract

The impact of center volume on outcomes in pediatric hematopoietic cell transplantation (HCT) is not well established. We retrospectively analyzed data from a nationwide registry, including 6966 pediatric patients who underwent their first allogeneic HCT at 123 centers in Japan between 2001 and 2020. Centers were categorized by transplant volume as low volume centers (C1, the smallest number of transplantation), medium-low volume centers (C2), medium-high volume centers (C3), and high volume centers (C4, the greatest number of transplantation), and outcomes were compared across these categories. The analysis revealed no statistically significant differences in HCT outcomes among center categories. The 5-year OS by center category was 66.8% (95% CI 64.4–69.0%) for C1, 66.8% (95% CI 64.5–69.0%) for C2, 67.9% (95% CI 65.6–70.2%) for C3, and 68.3% (95% CI 65.9–70.6%) for C4. These results were consistent even when analysis was restricted to malignant and nonmalignant diseases. Our findings suggest that, unlike in adult HCT, outcomes for pediatric HCT are not significantly affected by center volume. These results indicate the consistent quality of care across centers, supporting the accessibility of HCT at various institutions for pediatric patients.

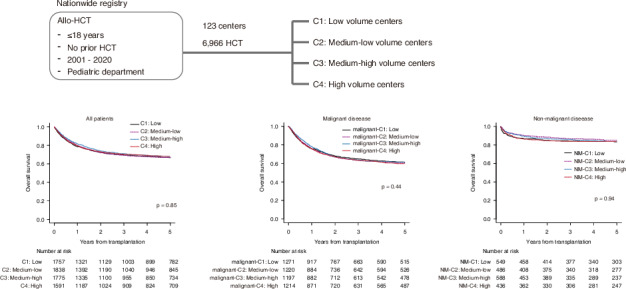

## Introduction

Hematopoietic cell transplantation (HCT) offers curative potential for conditions such as refractory leukemia [1, 2], bone marrow failure syndromes [3], immune deficiencies [4, 5], and inborn errors of metabolism [6]. However, intensive conditioning regimens and post-transplant immune responses often result in severe, sometimes life-threatening complications, necessitating effective management strategies to improve post-transplant survival.

Several factors influencing post-transplant survival are beyond the physician’s control, such as the disease type, and the amount of residual tumor. However, other factors, including conditioning regimen selection and donor matching, can be optimized. Supportive care is also crucial for safe transplantation. Institutional practices and physician experience often shape strategies for conditioning, immunosuppression, and the management of infections and organ dysfunction, potentially leading to variability in outcomes between centers.

The “center effect” has been documented in several studies [7–11], with some reports suggesting that high-volume centers may achieve better outcomes, though findings are inconsistent. High-volume centers might benefit from accumulated experience, enabling better management of complications and other factors collectively reduce transplant-related mortality. Conversely, high-volume centers may also treat patients with a higher relapse risk or more complex conditions. On the other hand, low-volume centers might provide more individualized care to their small number of transplant patients, potentially contributing to improved survival rates.

Most research on center effects has focused on adult patients. Pediatric transplantation differs in its indications and disease distribution, which include not only leukemia but also bone marrow failure, primary immune deficiencies, and inborn errors of metabolism. The accumulation of pediatric transplantation experience may follow a different trajectory than in adults, and findings from adult studies may not be directly extrapolatable to pediatric patients. Few studies have examined center effects in pediatric transplantation [12–15]. A report has indicated that centers with a high volume of haploidentical transplants showed better outcomes [15], whereas another study found no correlation between transplant center volume and outcomes in intensive care settings. However, these studies are often limited by sample size and disease scope.

Therefore, to assess the center effect in pediatric transplantation and provide insights for improving outcomes, we conducted an analysis using nationwide registry data.

## Methods

### Study population

All data were collected using the Transplant Registry Unified Management Program, sponsored by the Japanese Society for Transplantation and Cellular Therapy and the Japanese Data Center for Hematopoietic Cell Transplantation. This registration program covers over 99% of transplants nationwide [16].

To evaluate the treatment experience of pediatric patients, we included pediatric cases aged 19 years or younger at HCT, who received their first HCT from an allogeneic donor between 2001 and 2020. The transplant registry is organized by department, and cases are registered accordingly. Therefore, we included only those departments classified as pediatric departments in this analysis, even if the patients were under 19 years of age. Additionally, a small number of facilities operate joint teams comprising pediatric and adult departments; cases from such teams were excluded from this analysis. Allogeneic transplants for solid tumors were also excluded.

### Ethics approval and consent to participate

All study procedures complied with the Helsinki Declaration. The study was devised by the Complication Working Group of the Japanese Society for Transplantation and Cellular Therapy, and approved from the Data Management Committee of the Japanese Data Center for Hematopoietic Cell Transplantation (#20-70). The study was also approved by the Institutional Review Board of the University of Tokyo Hospital (#2022273NI). All patients provided informed consent for the use of their clinical data for research purposes.

### Statistical analysis

Center experience was defined based on the number of allogeneic HCT performed during the 20-year period, by which centers were divided into four groups using quartiles. Institutions were categorized as low volume centers (C1, the smallest number of transplantation), medium-low volume centers (C2), medium-high volume centers (C3), and high volume centers (C4, the greatest number of transplantation). For malignant/non-malignant specific analysis, institutions were re-categorized according to the number of transplantations performed for malignant diseases as low volume centers (malignant-C1/NM-C1, the smallest number of transplantation for malignant/non-malignant diseases), medium-low volume centers (malignant-C2/NM-C2), medium-high volume centers (malignant-C3/NM-C3), and high volume centers (malignant-C4/NM-C4, the greatest number of transplantation for malignant/non-malignant diseases).

The median follow-up time was estimated using the Reverse Kaplan-Meier method, where censoring events were treated as failures. The probability of overall survival (OS) was estimated using Kaplan-Meier methods. Cumulative incidence curves were used in a competing-risk setting to calculate the probability of non-relapse mortality and relapse. Multivariate analysis was performed using the Cox proportional-hazard regression model. A two-sided *p* value of less than 0.05 was considered to be significant. All statistical analyses were performed with EZR (Saitama Medical Center, Jichi Medical University, Saitama, Japan), which is a graphical user interface for R (The R Foundation for Statistical Computing, Vienna, Austria). More precisely, it is a modified version of R commander designed to add statistical functions frequently used in biostatistics [17]. Data preparation was conducted using a script provided by Drs. Yoshinobu Kanda and Junya Kanda [18].

## Results

### Transplantation and institutions

The study included 6966 patients who underwent transplantation at 123 centers (Table 1). C1 comprised centers performing ≤63 allo-HCT over 20 years (≤3.2 HCT/year), while C4 included centers performing ≥227 allo-HCT ( ≥ 11.4 HCT/year). Age distribution across categories is shown in Supplementary Fig. 1.

**Table 1:** Characteristics of patients according to the center volume.

	C1	C2	C3	C4	*P* value
Number of patients	1760	1839	1776	1591	
Number of institutions	86	20	11	6	
Number of HCT per institutions, range	1-63	67-119	120-213	227-351	
Age at HCT, median (range)	8 (0-19)	8 (0-19)	7 (0-19)	7 (0-19)	<0.001
Sex, n (%)					0.41
Female	713 (40.5)	740 (40.3)	676 (38.1)	645 (40.5)	
Male	1047 (59.5)	1098 (59.7)	1096 (61.9)	946 (59.5)	
Underlying disease, n (%)					<0.001
Malignant	1333 (75.7)	1399 (76.1)	1199 (67.5)	975 (61.3)	
Non-malignant	427 (24.3)	440 (23.9)	577 (32.5)	616 (38.7)	
Stem cell source, n (%)					<0.001
Matched related donor	427 (26.1)	397 (22.8)	328 (19.6)	282 (18.9)	
1-antigen mismatched related donor	142 (8.7)	122 (7)	125 (7.5)	113 (7.6)	
2-antigen mismatched related donor	75 (4.6)	121 (6.9)	110 (6.6)	89 (6)	
Unrelated donor	428 (26.1)	523 (30)	649 (38.7)	559 (37.4)	
Cord blood	565 (34.5)	580 (33.3)	463 (27.6)	450 (30.1)	
Conditioning, n (%)					<0.001
Myeloablative	1237 (71.9)	1210 (66.9)	1205 (68.9)	923 (59.0)	
TBI	732 (42.6)	816 (45.1)	680 (38.9)	492 (31.5)	
Non-TBI	505 (29.4)	394 (21.8)	525 (30.0)	431 (27.6)	
Reduced intensity	483 (28.1)	600 (33.1)	545 (31.1)	641 (41.0)	
Year of HCT, n (%)					0.005
2001–2010	930 (52.8)	935 (50.8)	833 (46.9)	801 (50.3)	
2011–2020	830 (47.2)	904 (49.2)	943 (53.1)	790 (49.7)	

Institutions were divided into four groups using quartiles. In order of decreasing number of transplants experienced during the 20-year period, Category 1 (C1, the smallest number of transplantation), Category 2 (C2), Category 3 (C3), and Category 4 (C4, the greatest number of transplantation). The years in which transplants were carried out were divided into two categories for analysis: before 2010 and after 2011.

*TBI* total body irradiation.

Centers with higher transplant volumes, such as C3 and C4, had a relatively higher proportion of non-malignant diseases, with fewer umbilical cord blood transplants and more non-myeloablative transplants.

### Outcome of HCT

**Fig. 1:**
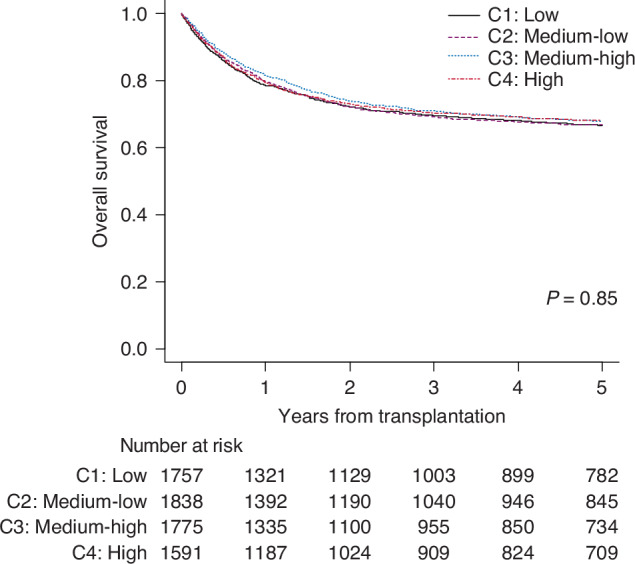
Overall survival according to the center category. Patients who underwent transplantation at low volume centers (C1, the smallest number of transplantation), medium-low volume centers (C2), medium-high volume centers (C3), and high volume centers (C4, the greatest number of transplantation) are compared.

The median follow-up period for surviving patients was 7.2 years, with an overall 5-year OS of 67.4% (95% confidence interval [CI]; 66.3–68.6%). The 5y-OS by center category was 66.8% (95% CI 64.4–69.0%) for C1, 66.8% (95% CI 64.5–69.0%) for C2, 67.9% (95% CI 65.6–70.2%) for C3, and 68.3% (95% CI 65.9–70.6%) for C4, with no statistically significant differences (p = 0.85) (Fig. 1). Early post-transplant survival rates did not consistently correlate with center experience. The 100-day OS was 90.9% (95% CI 89.4–92.1%) for C1, 92.1% (95% CI 90.8–93.3%) for C2, 92.9% (95% CI 91.6–94.0%) for C3, and 91.6% (95% CI 90.1–92.9%) for C4. Similar trends were observed for transplants conducted after 2011, with no significant differences in OS by center category (Supplementary Fig. 2).

No clear differences were observed in the distribution of causes of death across center categories (Supplementary Fig. 3). A small number of patients had late events at later than 10 years from HCT. Cause of these late death were predominantly secondary malignancies, with C3 and C4 including patients with underlying genetic diseases such as Fanconi anemia.

The incidence of grade II–IV acute GVHD at day 100 was 35.0% (95% CI, 32.7–37.3%) for C1, 35.5% (95% CI 33.2–37.7%) for C2, 34.8% (95% CI 32.5–37.0%) for C3, and 36.8% (95% CI 34.4–39.1%) for C4 (p = 0.45) (Supplementary Fig. 4A). The incidence of grade III–IV acute GVHD at day 100 was 13.8% (95% CI 12.2–15.5%) for C1, 15.6% (95% CI 14.0–17.3%) for C2, 14.5% (95% CI 12.8–16.2%) for C3, and 14.9% (95% CI 13.2–16.7%) for C4 (p = 0.56). The incidence of chronic GVHD at 1 year was 23.4% (95% CI 21.4–25.5%) for C1, 20.1% (95% CI 18.3–22.1%) for C2, 20.1% (95% CI 18.2–22.1%) for C3, and 17.7% (95% CI 15.8–19.7%) for C4 (p < 0.001) (Supplementary Fig. 4B).

### Outcomes of HCT for malignant diseases

**Fig. 2:**
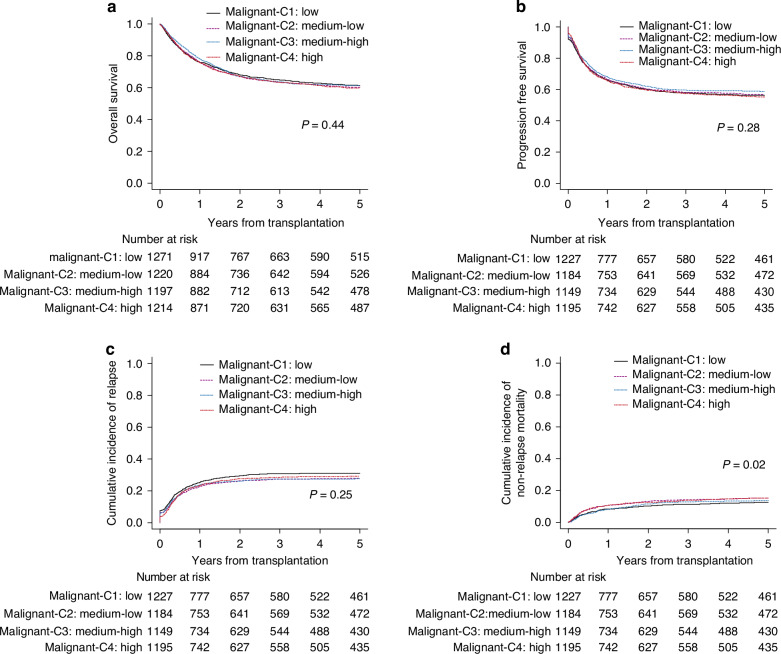
Overall survival of transplantation for malignant diseases according to the center category. Patients who underwent transplantation at low volume centers (malignant-C1, the smallest number of transplantation), medium-low volume centers (malignant-C2), medium-high volume centers (malignant-C3), and high volume centers (malignant-C4, the greatest number of transplantation) are compared. **a** Overall survival, (**b**) disease free survival, (**c**) cumulative incidence of relapse, and (**d**) cumulative incidence of non-relapse mortality.

In an analysis restricted to malignant diseases (Supplementary Table 1), no statistically significant differences in OS were observed between center categories. The 5-year OS was 61.4% (95% CI, 58.5–64.2%) for malignant-C1, 60.2% (95% CI, 57.3–63.0%) for malignant-C2, 61.3% (95% CI, 58.3–64.1%) for malignant-C3, and 59.7% (95% CI, 56.7–62.5%) for malignant-C4 (p = 0.44) (Fig. 2a). The 5-year disease free survival was 56.4% (95% CI, 53.5–59.2%) for malignant-C1, 56.8% (95% CI, 53.8–59.6%) for malignant-C2, 58.8% (95% CI, 55.8–61.6%) for malignant-C3, and 55.3% (95% CI, 52.4–58.2%) for malignant-C4 (p = 0.28) (Fig. 2b).

The 5-year cumulative incidence of relapse was 31.1% (95% CI, 28.4–33.7%) for malignant-C1, 27.9% (95% CI, 25.3–30.5%) for malignant-C2, 27.6% (95% CI, 25.0–30.3%) for malignant-C3, and 29.4% (95% CI, 26.7–32.0%) for malignant-C4 (p = 0.25) (Fig. 2c). The 5-year cumulative incidence of non-relapse mortality was 12.6% (95%CI, 10.7–14.6%) for malignant-C1, 15.4% (95%CI, 13.3–17.5%) for malignant-C2, 13.6% (95%CI, 11.6–15.7%) for malignant-C3, and 15.3% (95%CI, 13.3–17.5%) for malignant-C4 (p = 0.02) (Fig. 2d).

When analyzing data restricted to patients with HCT for CR1 of ALL or AML, the survival curves were nearly identical across center categories (Supplementary Fig. 5). The 5-year OS was 73.6% (95%CI, 68.7–77.9%) for malignant-C1, 78.1% (95%CI, 73.6–81.9%) for malignant-C2, 77.7% (95%CI, 73.0–81.6%) for malignant-C3, and 74.7% (95%CI, 69.8–78.9%) for malignant-C4 (p = 0.72). The 5-year cumulative incidence of relapse was 19.9% (95%CI, 16.0–24.2%) for malignant-C1, 18.6% (95%CI, 14.9–22.7%) for malignant-C2, 18.9% (95%CI, 15.0–23.1%) for malignant-C3, and 21.0% (95%CI, 17.0–25.4%) for malignant-C4 (p = 0.84). The 5-year cumulative non-relapse mortality was 11.5% (95%CI, 8.4–15.1%) for malignant-C1, 8.7% (95%CI, 6.1–11.7%) for malignant-C2, 9.0% (95%CI, 6.3–12.2%) for malignant-C3, and 9.1% (95%CI, 6.4–12.3%) for malignant-C4 (p = 0.74).

### Outcomes of HCT for non-malignant diseases

An analysis focusing on non-malignant diseases (Supplementary Table 2) showed that bone marrow failure syndromes were the most common diagnosis in all centers. Metabolic disorders were slightly less frequent in NM-C1, and Fanconi anemia patients were transplanted more commonly transplanted in NM-C4.

**Fig. 3:**
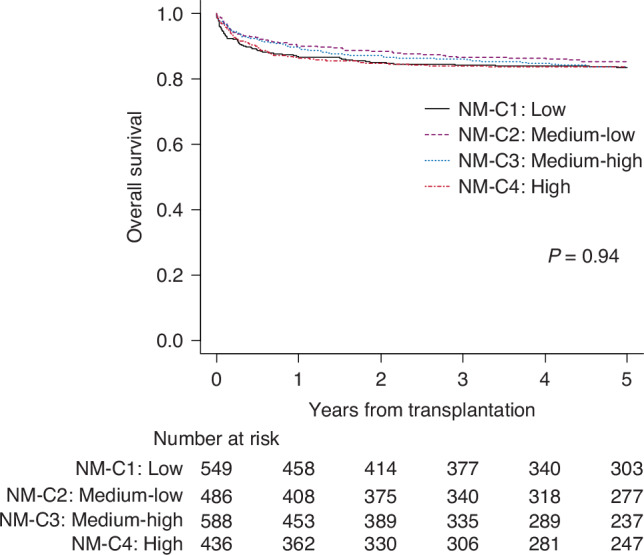
Overall survival of transplantation for non-malignant diseases according to the center category. Patients who underwent transplantation at low volume centers (NM-C1, the smallest number of transplantation), medium-low volume centers (NM-C2), medium-high volume centers (NM-C3), and high volume centers (NM-C4, the greatest number of transplantation).

Even when focusing on non-malignant diseases, no significant differences in OS were observed across center categories. The 5-year OS was 90.2% (95% CI 85.3–93.5%) for NM-C1, 90.7% (95% CI 86.2–93.8%) for NM-C2, 92.3% (95% CI 88.3–95.0%) for NM-C3, and 89.2% (95% CI 84.3–92.7%) for NM-C4 (p = 0.94) (Fig. 3).

### Multivariate analysis for outcomes

Multivariate analysis focusing on both acute leukemia (Table 2) and non-malignant diseases (Table 3) showed no significant center effect on post-transplant survival. Differences in transplant outcomes may vary depending on the cell source. As donor selection criteria are often shaped by transplant experience, a potential center effect cannot be ruled out. However, in our study, the influence of transplant experience on outcomes was not evident, even after performing a multivariate analysis that accounted for cell source.

**Table 2:** Multivariate analysis on overall mortality in transplantation for acute lymphoblastic leukemia and acute myeloid leukemia.

	Hazard ratio (95% CI)	*P* value
Center volume		
2^nd^ vs 1^st^	0.95 (0.83–1.09)	0.43
3^rd^ vs 1^st^	1.07 (0.93–1.23)	0.35
4^th^ vs 1^st^	1.08 (0.93–1.25)	0.33
Age		
<10 vs. ≥10	0.84 (0.76–0.93)	<0.01
Donor		
Related donor vs unrelated donor	1.24 (1.09–1.41)	<0.01
Cord blood vs unrelated donor	1.23 (1.08–1.42)	<0.01
Disease		
AML vs ALL	0.90 (0.81–1.00)	0.42
Disease status		
CR2 vs CR1	1.73 (1.49–2.00)	<0.01
Others vs CR1	4.02 (3.55–4.55)	<0.01
Transplantation year		
2011- vs -2010	0.69 (0.62–0.77)	<0.01

*AML* acute myeloid leukemia, *ALL* acute lymphoblastic leukemia, *CR1* first complete remission, *CR2* second complete remission.

**Table 3:** Multivariate analysis on overall mortality in transplantation for non-malignant diseases.

	Hazard ratio (95% CI)	*P* value
Center volume		
2^nd^ vs 1^st^	0.81 (0.58–1.13)	0.23
3^rd^ vs 1^st^	0.84 (0.61–1.15)	0.28
4^th^ vs 1^st^	0.94 (0.70–1.27)	0.69
Age		
<10 vs. ≥10	0.78 (0.62–0.99)	<0.04
Donor		
Related donor vs unrelated donor	0.85 (0.64–1.12)	<0.26
Cord blood vs unrelated donor	1.59 (1.20–2.10)	<0.01
Disease		
PID vs. BMF	1.95 (1.45–2.61)	<0.01
Others vs. BMF	2.15 (1.61–2.88)	<0.01
Transplantation year		
2011- vs -2010	0.76 (0.61–0.90)	<0.04

*PID* primary immunodeficiency, *BMF* bone marrow failure.

## Discussion

This study did not find substantial differences in survival outcomes between high- and low-volume centers for pediatric HCT. While the study does not prove the absence of differences, and may be subject to various potential biases due to a retrospective nature, it does suggest that clinically significant differences are unlikely. These results were consistent even when the analysis was restricted to malignant or non-malignant diseases or more recent transplants.

In contrast, several studies on adult HCT have reported a center effect, including studies using the same registry data [9, 11]. The acute non-relapse mortality may reflect transplant quality. However, even when focusing on early post-transplant cumulative mortality (e.g., within 100 days), no evidence suggested that high-volume centers performed better.

Given the smaller number of pediatric HCT compared to adults, one interpretation is that pediatric transplants are managed at a comparable quality across low- and high-volume centers. While many hospitals treat pediatric hematologic and oncologic diseases, only a limited number of facilities perform allo-HCT, contributing a certain level of quality of transplantation. Additionally, the rapid dissemination of information in recent years may have contributed to standardizing care across centers. It is crucial to rigorously assess and communicate the transplantation experience from each facility as evidence, rather than allowing them to remain anecdotal.

However opposite perspective is also valid. The relatively small number of pediatric allo-HCT, even at high-volume centers suggested that center experience was still insufficient for pediatric transplantation. It may be more important to centralize more patients than at present in order to accumulate experience and improve transplantation for rare pediatric patients. Especially, it is important to acknowledge that transplantation for rare diseases require unique management skill and knowledge [19]. Additionally, rare and severe transplantation-related complications may not be encountered without a large volume of transplants. Given the limited evidence available in rare diseases and complications, a centralized approach should be preferable to enhance evidence generation. Furthermore, conducting transplants at centers that manage similar diseases can provide valuable peer support for patients and their families. The approach to transplantation should therefore be tailored, balancing centralization and decentralization according to the specific needs of each disease.

Our study has several limitations, the most important being its retrospective design, which introduce biases affecting post-transplant outcomes. However, it is practically impossible to control for the center effect in a prospective interventional study. We attempted to mitigate this limitation by increasing the number of cases analyzed. While differences may exist in more challenging transplants, such as second transplants, these were not included in the current analysis. Such cases are few in number and require innovative approaches to study. However, it can be assumed that facilities with extensive transplant experience tend to perform a higher number of second and third transplants. This study primarily assessed overall survival. However, post-transplant, patients may experience significant complications, some of which are late-onset and have a profound impact on quality of life, such as chronic GVHD. The frequency and severity of complications were only partially analyzed in this study.

In conclusion, our analysis suggests that there are no substantial differences in survival outcomes between high- and low-volume centers for pediatric transplants, indicating that consistent quality of care is maintained across centers. This is positive information for patients, and it is recommended that pediatric patients undergo transplants at the most accessible center.

## Supplementary information

Supplementary Information

## Data Availability

The data of this study are not publicly available due to ethical restrictions that it exceeds the scope of the recipient/donor’s consent for research use in the registry. Data may be available from the corresponding author upon reasonable request and with permission of the JSTCT/JDCHCT.
