# Supplementary material for: Impact of center volume on outcomes in allogeneic hematopoietic cell transplantation for children
Source: Bone Marrow Transplant. 2025 Apr 10;60(6):851–6. doi: 10.1038/s41409-025-02569-3 (PMC12151867; doi:10.1038/s41409-025-02569-3)
Supplement: Supplementary file 1 — Supplementary Information [file 41409_2025_2569_MOESM1_ESM.pdf]

Supplementary Table 1. Characteristics of patients according to the center volume focusing on transplantation for malignant diseases

Volume	Malignant-C1	Malignant-C2	Malignant-C3	Malignant-C4
Number of patients	1333	1399	1199	975
Median age at HCT	8	9	8	7
Underlying disease				
ALL	616 (46.2)	674 (48.2)	591 (49.3)	447 (45.8)
CR1	260 (42.2)	294 (43.6)	273 (46.2)	167 (37.4)
CR2	155 (25.2)	159 (23.6)	151 (25.5)	127 (28.4)
Others	201 (32.6)	221 (32.8)	167 (28.3)	153 (34.2)
AML	359 (26.9)	400 (28.6)	365 (30.4)	265 (27.2)
CR1	154 (42.9)	163 (40.8)	168 (46)	97 (36.6)
CR2	63 (17.5)	78 (19.5)	82 (22.5)	47 (17.7)
Others	142 (39.6)	159 (39.8)	115 (31.5)	121 (45.7)
Others	358 (26.9)	325 (23.2)	243 (20.3)	263 (27)

Institutions are re-categorized according to the number of transplantations performed for malignant diseases as low volume centers (malignant-C1, the smallest number of transplantation), medium-low volume centers (malignant-C2), medium-high volume centers (malignant-C3), and high volume centers (malignant-C4, the greatest number of transplantation).

ALL, acute lymphoblastic leukemia; AML, acute myeloid leukemia; CR1, first complete remission; CR2, second complete remission.

Supplementary Table 2. Characteristics of patients according to the center volume focusing on transplantation for non-malignant diseases

Volume	NM-C1	NM-C2	NM-C3	NM-C4
Number of patients	549	486	589	436
Median age at HCT	6	7.5	5	8
Underlying disease				
Bone marrow failure	307 (55.9)	262 (53.9)	221 (37.5)	219 (50.2)
Fanconi anemia	16 (2.9)	20 (4.1)	17 (2.9)	57 (13.1)
Primary immunodeficiency	108 (19.7)	98 (20.2)	251 (42.6)	70 (16.1)
Inborn error of metabolism	37 (6.7)	54 (11.1)	57 (9.7)	55 (12.6)
Others	97 (17.7)	72 (14.8)	60 (10.2)	92 (21.1)

Institutions are re-categorized according to the number of transplantations performed for non-malignant diseases as low volume centers (NM-C1, the smallest number of transplantation), medium-low volume centers (NM-C2), medium-high volume centers (NM-C3), and high volume centers (NM-C4, the greatest number of transplantation).

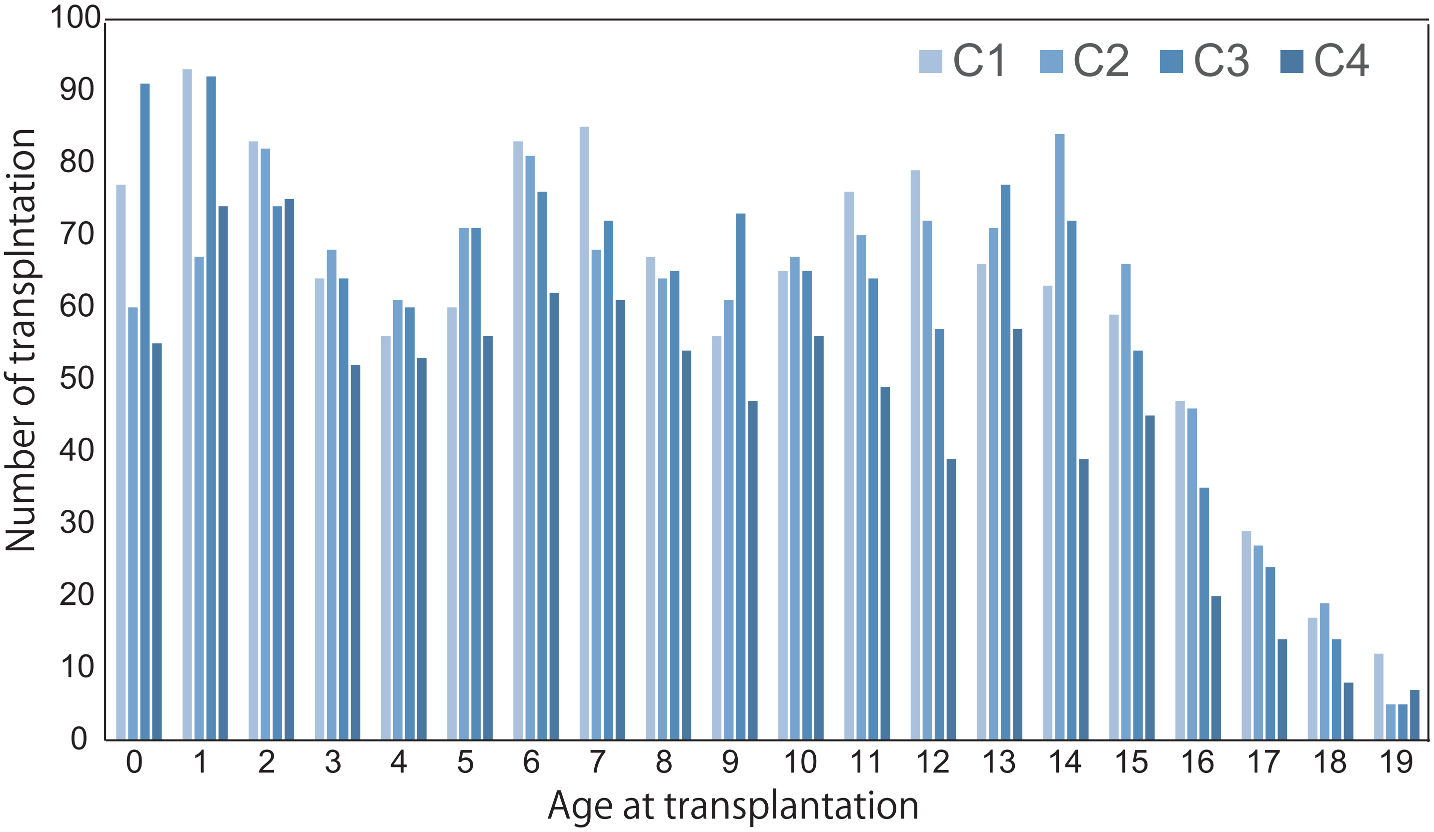

Supplementary Figure 1. Number of transplantation according to age

The age distribution of the number of transplants over the 20-year period is shown for each facility category. Institutions were categorized as low volume centers (C1, the smallest number of transplantation), medium-low volume centers (C2), medium-high volume centers (C3), and high volume centers (C4, the greatest number of transplantation). The number of patients aged 20 years or older (excluded from the outcome analysis) is as follows: 33 patients in C1, 40 patients in C2, 44 patients in C3, and 62 patients in C4.

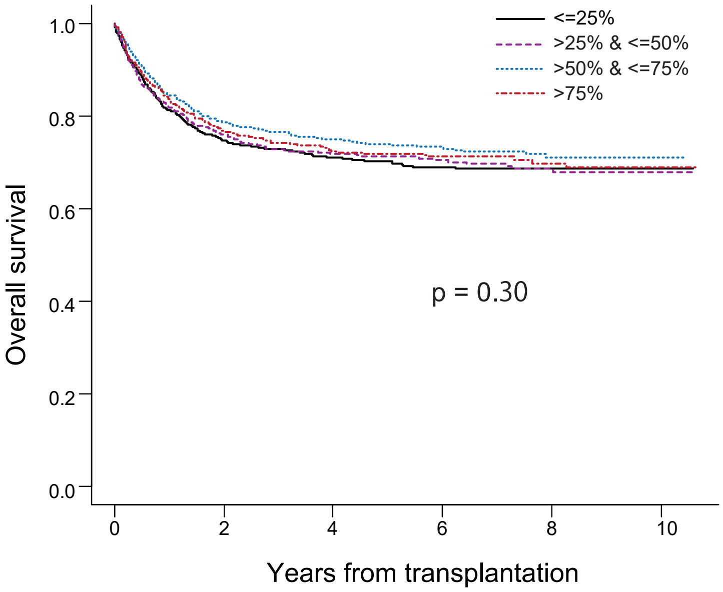

Number at risk

<=25%	906	550	374	233	113	15
>25% & <=50%	849	524	364	220	97	12
>50% & <=75%	850	515	353	219	98	9
>75%	862	504	324	181	86	11

Supplementary Figure 2. Overall survival of transplantation after 2011

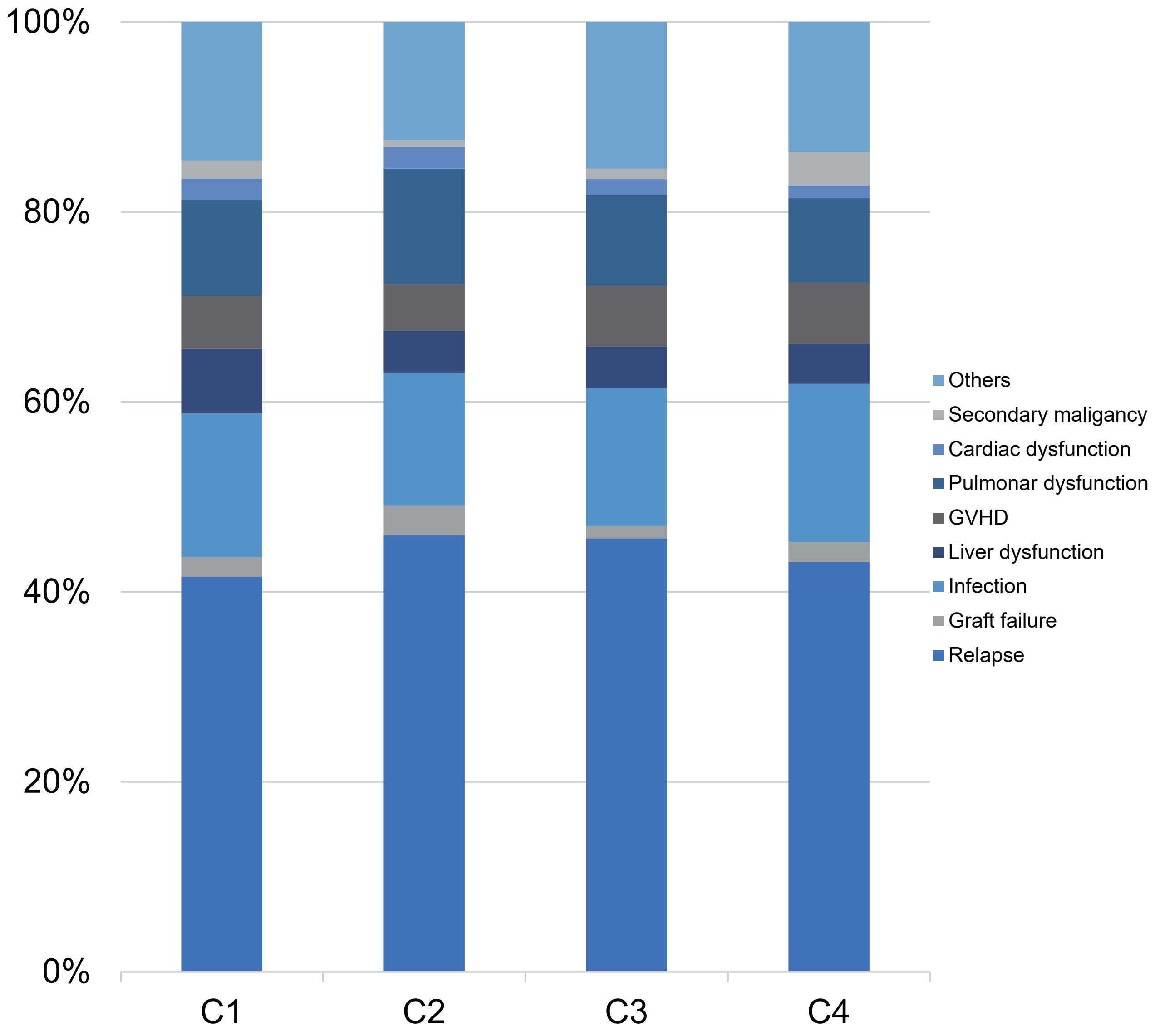

Supplementary Figure 3. Distribution of cause of death

The distribution of main cause of death is shown by center category. Liver disfunction includes hepatic veno-occlusive diseaes/sinusoidal obstruction syndromme. Pulmonary infection is categorized as “infection” .

A)

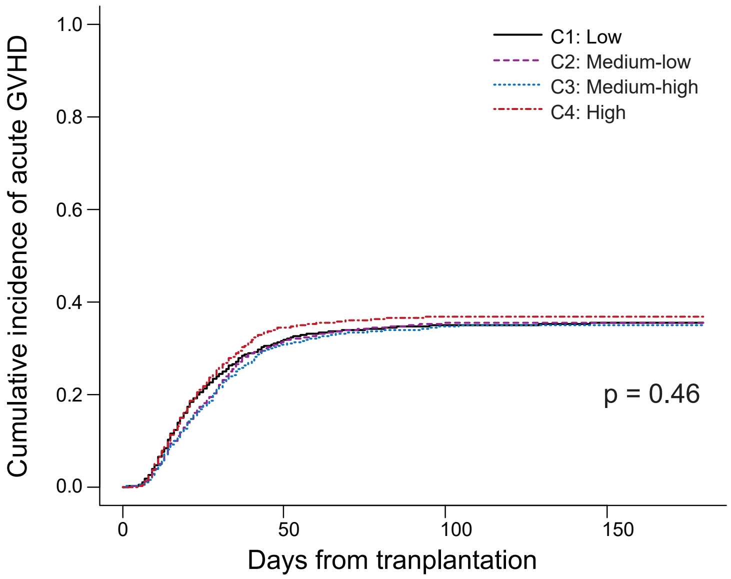

Number at risk

C1: Low	1694	1030	909	849
C2: Medium-low	1795	1107	965	905
C3: Medium-high	1736	1108	963	896
C4: High	1565	926	824	787

B)

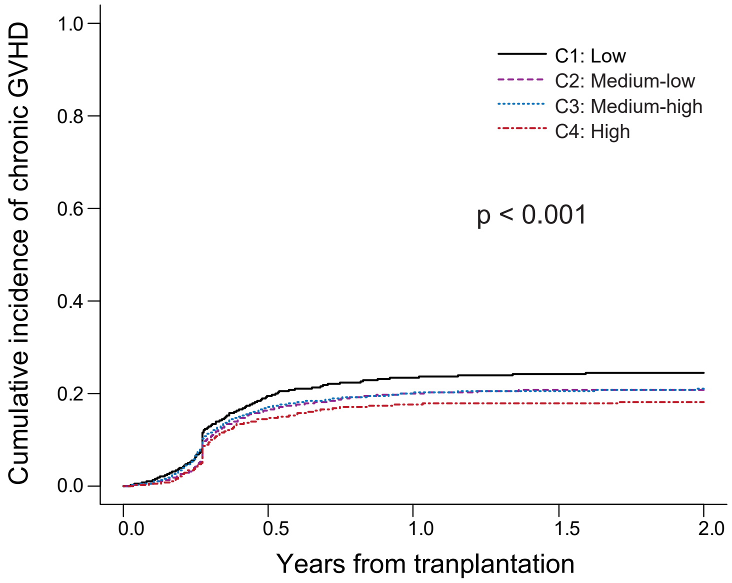

Number at risk

C1: Low	1697	1126	935	842	775
C2: Medium-low	1786	1232	1042	944	882
C3: Medium-high	1726	1200	1008	902	823
C4: High	1551	1091	922	846	789

Supplementary Figure 4. Cumulative incidences of GVHD according to the center category
Cumulative incidences of grade II–IV acute GVHD (A) and chronic GVHD (B) are shown.

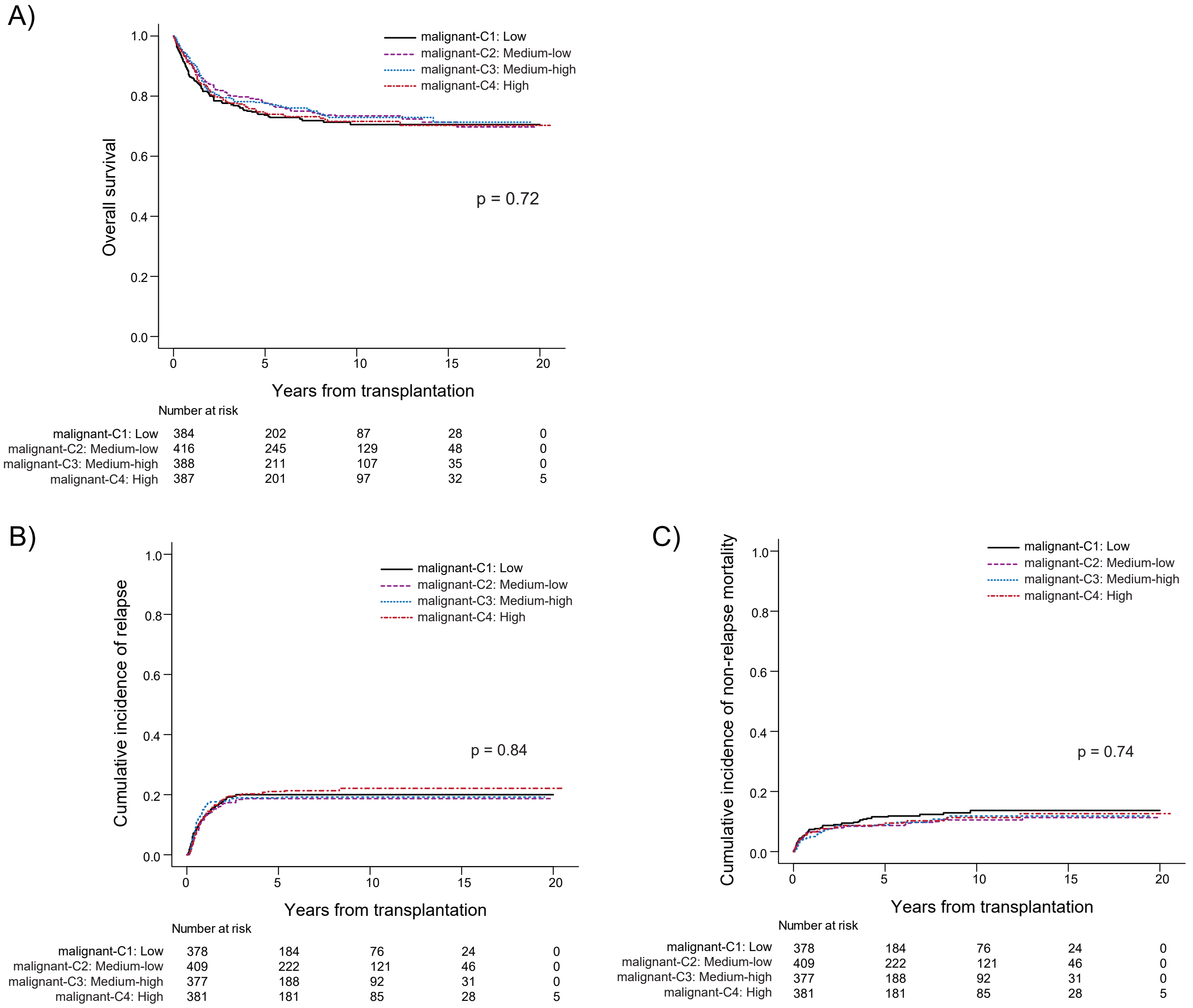

Supplementary Figure 5. Outcomes of transplantation for leukemia in first remission according to the center category

A) Overall survival, B) cumulative incidence of relapse, and C) cumulative incidence of non-relapse mortairty of transplantation for acute lymphoblastic leukemkia and acute myeloid leukemia in their first remission are shown.
